# mRNA-Associated Processes and Their Influence on Exon-Intron Structure in *Drosophila melanogaster*

**DOI:** 10.1534/g3.116.029231

**Published:** 2016-03-28

**Authors:** Gildas Lepennetier, Francesco Catania

**Affiliations:** Institute for Evolution and Biodiversity, University of Münster, 48149, Germany

**Keywords:** gene structure, capping, telescripting, cleavage and polyadenylation, *Drosophila*

## Abstract

mRNA-associated processes and gene structure in eukaryotes are typically treated as separate research subjects. Here, we bridge this separation and leverage the extensive multidisciplinary work on *Drosophila melanogaster* to examine the roles that capping, splicing, cleavage/polyadenylation, and telescripting (*i.e*., the protection of nascent transcripts from premature cleavage/polyadenylation by the splicing factor U1) might play in shaping exon-intron architecture in protein-coding genes. Our findings suggest that the distance between subsequent internal 5′ splice sites (5′ss) in *Drosophila* genes is constrained such that telescripting effects are maximized, in theory, and thus nascent transcripts are less vulnerable to premature termination. Exceptionally weak 5′ss and constraints on intron-exon size at the gene 5′ end also indicate that capping might enhance the recruitment of U1 and, in turn, promote telescripting at this location. Finally, a positive correlation between last exon length and last 5′ss strength suggests that optimal donor splice sites in the proximity of the pre-mRNA tail may inhibit the processing of downstream polyadenylation signals more than weak donor splice sites do. These findings corroborate and build upon previous experimental and computational studies on *Drosophila* genes. They support the possibility, hitherto scantly explored, that mRNA-associated processes impose significant constraints on the evolution of eukaryotic gene structure.

Protein-coding genes in eukaryotes have a paradoxical structure. They contain sequences that need to be removed from precursor messenger (pre-m)RNAs to enable the synthesis of functional proteins. These intragenic noncoding sequences, known as spliceosomal introns (Berget *et al.* 1977; Chow *et al.* 1977; Evans *et al.* 1977; Goldberg *et al.* 1977) are excised by the spliceosome (Will and Luhrmann 2011), a nonpreassembled nuclear machinery that consists of five uridine-rich small nuclear RNAs (U1, U2, U4, U5, and U6) and hundreds of accessory proteins (Lamond 1993; van der Feltz *et al.* 2012).

While several genetic and epigenetic factors influence intron recognition and splicing (Luco *et al.* 2010; Caceres and Hurst 2013; Ye *et al.* 2014), three signals typically define an intron and are crucial for its excision: the 5′ splice site (5′ss), the 3′ splice site (3′ss), and the branch site (Seraphin and Rosbash 1989; Umen and Guthrie 1995; Du and Rosbash 2002). The 5′ss, also known as the donor splice site, is located at the very beginning of an intron, whereas the 3′ss (or acceptor splice site) marks its end. The branch site may be found 20–40 nt before the intron 3′ end (Lamond 1993). These splicing signals are recognized and bound by distinct small nuclear ribonucleoproteins (snRNPs), one of which, the 5′ss-binding U1 snRNP (hereafter U1), triggers spliceosome assembly (Kondo *et al.* 2015). The fidelity of the interactions between snRNPs and splicing signals respects RNA–RNA base-pairing complementarity, and is critical for a correct splicing (Fox-Walsh and Hertel 2009). Therefore, sequence complementarity is typically employed as a quality measure for splicing signals, particularly for the 5′ss and the 3′ss (Yeo and Burge 2004). More specifically, high-fidelity, or strong, splice sites are characterized by high levels of sequence complementarity, whereas weak splice sites increase the probability of splicing failure, all else being equal (Fox-Walsh and Hertel 2009; Roca *et al.* 2013).

RNA splicing is not an isolated process in the cell. Rather, it interacts tightly with several co- and post-transcriptional processes, including mRNA capping, and 3′ end cleavage/polyadenylation, among others. It is well established that these molecular interactions are instrumental for the accurate processing of nascent transcripts (Maniatis and Reed 2002; Bentley 2014). What is less clear is whether these interactions affect the structure of eukaryotic genes, as has been previously proposed (Catania and Lynch 2008; Catania and Lynch 2013). A number of experimental studies provide clues as to how mRNA-associated processes might influence gene architecture.

First, empirical evidence shows that at the pre-mRNA 5′ end the cap-binding complex (CBC) enhances the association between U1 and the cap-proximal 5′ss in human and yeast (Izaurralde *et al.* 1994; Colot *et al.* 1996; Lewis *et al.* 1996; Gornemann *et al.* 2005; Qiu *et al.* 2007; Pabis *et al.* 2013). This suggests that splicing might be facilitated at the mRNA 5′ end and that, consequentially, introns may accumulate preferentially at this location (Sakurai *et al.* 2002; Lin and Zhang 2005; Ruvinsky and Ward 2006).

Second, it has been demonstrated (in human, mouse, and *Drosophila*) that 5′ss-bound U1 protects pre-mRNAs from premature cleavage/polyadenylation at nearby polyadenylation signals (PAS) (Ashe *et al.* 1997; Gunderson *et al.* 1998; Vagner *et al.* 2000; Kaida *et al.* 2010; Guo *et al.* 2011; Andersen *et al.* 2012). The protective effects of U1, termed telescripting, may extend to a median distance of ∼500 nt downstream from the bound 5′ss in *Drosophila*, and regulate the length of nascent gene transcripts in a U1 concentration-dependent manner (Berg *et al.* 2012). These observations suggest that the action range of telescripting might impose constraints on the distance between two subsequent 5′ss so as to minimize the risk of premature cleavage/polyadenylation within that interval.

Finally, while the effective recognition of the last-exon PAS is known to facilitate the splicing of the 3′-most intron (Niwa and Berget 1991; Rigo and Martinson 2008), it has been also demonstrated in yeast that splicing is disfavored in the proximity of the 3′-end termination signals (Tardiff *et al.* 2006). These findings suggest that the distance between splice sites and downstream PAS at the pre-mRNA tail might not be random.

Here, we draw from the wealth of knowledge on *Drosophila melanogaster* genetics, molecular and cell biology, and biochemistry, to generate and test three hypotheses. First, if the CBC truly helps the recruitment of U1 at the pre-mRNA 5′ end then we might detect distinct structural and/or sequential properties at the gene 5′ end that enable or result from these effects. Second, sequence motifs recruiting U1 might be distributed along eukaryotic genes such that the telescripting effects are maximized and, thus, the risk of premature cleavage/polyadenylation is minimized. Third, last introns and last exons might have specific properties that allow splicing and mRNA 3′-end termination to coexist at the pre-mRNA tail.

The patterned variations in intron and exon size, in splicing strength, and in the degree of DNA strand asymmetry of the polyadenylation AATAAA motif that we detect along the protein-coding genes of *D. melanogaster*—and of its distant relative *D. yakuba*—suggest that mRNA-associated processes might indeed influence exon-intron architecture in *Drosophila*.

## Materials and Methods

### Dataset

We built our dataset using the release 82 of Ensembl *D. melanogaster* genome and annotation (dos Santos *et al.* 2015): ftp://ftp.ensembl.org/pub/release-82/fasta/drosophila_melanogaster/dna/Drosophila_melanogaster.BDGP6.dna.toplevel.fa.gz and ftp://ftp.ensembl.org/pub/release-82/gtf/drosophila_melanogaster/Drosophila_melanogaster.BDGP6.82.gtf.gz.

We extracted information about gene structure using in-house python (python.org), bash (shell language), and R scripts (r-project.org). We randomly selected one isoform (rather than focusing only on the longest isoforms), and considered only protein-coding genes. Our dataset includes constitutively and alternatively spliced introns that contain no nested genes and whose size is equal to, or greater than, 32 nt (this operation allows us to minimize bias in motif count or double-counting the same sites from the procedure we used for scoring splice site strength; see below). Finally, to focus on RNA splicing mediated by the major spliceosome, we discarded introns with noncanonical splice sites (*i.e.*, no GT at the 5′ss or AG at the 3′ss). Our working dataset contains 7256 protein-coding genes with at least two introns (out of 13,900 extracted protein-coding genes), for a total of 35,896 introns.

### Splice site strength

In this study, splice site strength was measured as the degree of sequence similarity between spliceosomal small RNAs units and splice sites. We estimated the strength of 5′ss and 3′ss using the MaxEntScan scoring method (Yeo and Burge 2004), retrained with *D. melanogaster* splice site sequences as in McManus *et al.* (2014).

### Sliding-window analyses

We used a sliding-window approach to illustrate the trend of the examined correlations, similar to that described in Farlow *et al.* (2012). Briefly, after ranking the data according to the values of a particular variable (*e.g.*, first exon length), we estimated subsequent medians of 2000 observations for the remaining variables, using a step size of 1. So, each point on the graph is supported by exactly 2000 observations. Sliding-window analyses were conducted using default functions (rollapply, in the package zoo) in R. All the reported correlation coefficients are based on raw data.

### DNA strand asymmetry

We estimated the degree of DNA strand asymmetry (DSA) using published protocols (Zhang *et al.* 2008; Farlow *et al.* 2012). Chargaff second parity rule predicts that the number of mono- and oligo-nucleotides on one strand of DNA (*e.g.*, the sense DNA strand, Ns) is equal to the number of their reverse complements on the opposite strand (the anti-sense DNA strand, Na). The degree of DSA supplies a measure of the selective pressure exerted on a motif of a particular DNA strand (Mitchell and Bridge 2006). We studied the DSA for the classic polyadenylation motif AATAAA (Beaudoing *et al.* 2000), and for the 5′ splice site-like motif GGTAAG (Mount *et al.* 1992). Additionally, we examined the degree of DSA for the hexamers ATTAAA, AATATA, and TATAAA, which are putatively weaker PAS compared to the canonical AATAAA (Retelska *et al.* 2006). Finally, we used two anagrams of the motif AATAAA (TAAAAA and AAAAAT) to rule out that the DSA values observed for AATAAA within introns are an artifact of an underlying (di)nucleotide asymmetry. When conducting the DSA analyses, we studied exons and introns after discarding nucleotides that are potential sources of bias, *i.e.*, 3 nt at the exon’s 5′ and 3′ end, 6 nt at the 5′ end, and 40 nt at the 3′ end of introns. We used Equation 1 to calculate the DNA strand asymmetry of each of these motifs along introns or exons, where *S* is the asymmetry score, and *Ns* and *Na* are the counts of the motif under study in the sense and the antisense strand, respectively:

S=(Ns-Na)/(Ns+Na). (1)

### Nonrandom association of introns and their next exon

We tested the hypothesis that the size of the introns at specific positions (first, internal, or last), and that of their next exon, are nonrandomly associated. To this end, we first partitioned our dataset into three groups, *i.e.*, 5′ end, internal, and 3′ end regions. For each of these groups, we compared the size distribution of genuine introns plus their next exon units, with 100,000 distributions generated by randomly sampling (with replacement) equivalent numbers of introns and exons. Any intron in a group had the same probability to be chosen, and to be associated with any following (second) exon. A nonparametric two-sample Kolmogorov-Smirnov test was used to measure the level of discrepancy between the actual and the random size distributions. Kolmogorov-Smirnov *P*-values were estimated for each of the 100,000 simulations. An overall unique *P*-value was estimated as *N*/100,000, *i.e.*, the number of times that the test was nonsignificant (*P*-value < 0.05) divided by the number of simulations.

### Data availability

The authors state that all data necessary for confirming the conclusions presented in the article are represented fully within the article. All of the scripts are publicly available through GitHub at https://github.com/GildasLepennetier/GildasSources.

## Results

### Weak 5′ splice sites populate the gene 5′ end: a footprint of splicing-enhancing effects by the cap-binding complex?

At the pre-mRNA 5’ end, the CBC enhances the association of U1 with the cap-proximal 5′ss (Lewis *et al.* 1996). If splicing is enhanced at the 5’ end of pre-mRNAs, then it is possible that introns that are positioned near the CBC might not need to evolve (or maintain) a strong donor splice site.

**Figure 1:**
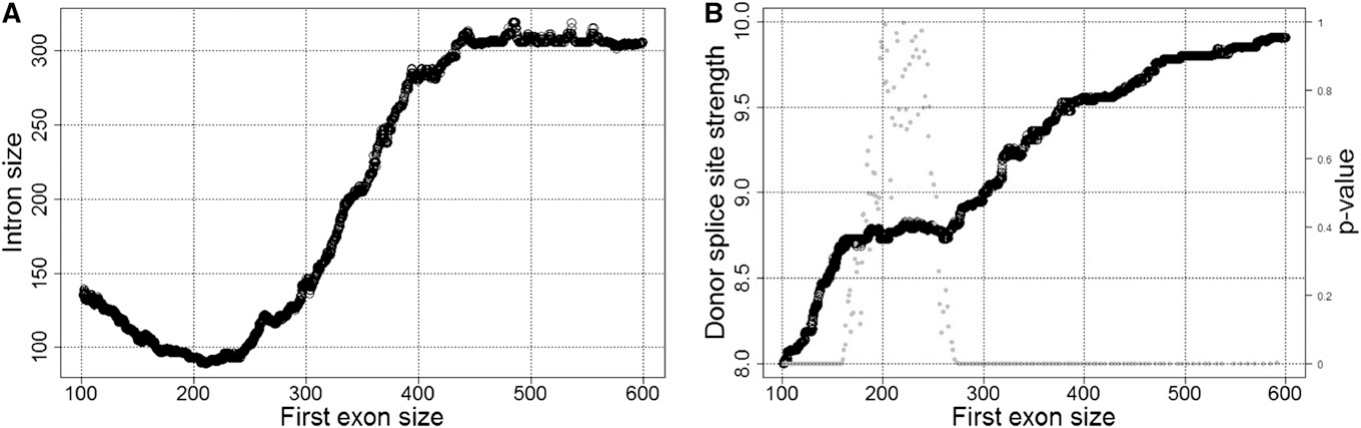
Relationship between first exon size (in nucleotides) and (A) first intron size or (B) strength of first-intron donor splice site. Data were ranked according to the X-axis variable and subsequent medians of 2000 observations (step size of 1) for *x*- and *y*-axis variables were estimated and plotted. B also shows the variations in the statistical significance of the positive association between the strength of the first donor splice site and its distance from the CBC (approximated by the first exon size). *P* values are represented by gray dots.

We found that the 5′ss of first introns in *D. melanogaster* are indeed significantly weaker compared to their counterparts in internal and last introns (Kruskal-Wallis test, *P* < 0.001, Table 1). To our knowledge, this observation has not been reported before, and, taken at face value, it is unexpected. First introns in *D. melanogaster* are larger, on average, compared to internal and last introns (Hong *et al.* 2006; Bradnam and Korf 2008) (Kruskal-Wallis test, *P* < 0.001; Table 1), and stronger signals are typically required for the accurate removal of large introns (Dewey *et al.* 2006; Fahey and Higgins 2007; Farlow *et al.* 2012). Our own observations confirm that intron size and splicing strength are coupled in *D. melanogaster* when all of the introns in our dataset are considered (35,896 observations; 5′ss: Kendall’s tau = 0.12; 3′ss: Kendall’s tau = 0.07; *P* < 0.001). Separate analyses of first, internal, and last introns, however, reveal that the positive relationship between 5′ss strength and intron size is relatively weak, though statistically significant, at the gene 5′ end (Supplemental Material, Table S1). Moreover, the association between first intron size and the 5′ most 5′ss even becomes negative when introns are near the CBC (*e.g.*, within the first 200 nt from the CBC: 2931 observations; Kendall’s tau = –0.04, *P* < 0.001) (Figure 1A). Although we cannot rule out alternative explanations, these findings lend support to the reported CBC-mediated enhanced recruitment of U1 at the first donor splice site.

**Table 1:** Summary statistics on the surveyed *D. melanogaster* introns partitioned according to their relative intragenic position

	First	Internal	Last
Number of observations	6967	19,504	7130
Average (median) intron size	1550 (145)	1092 (69)	392 (65)
Average (median) exon size	341 (233)	385 (212)	848 (596)
Average (median) 5′ss strength	8.6 (8.9)	9.3 (9.8)	9.3 (9.8)
Average (median) 3′ss strength	9.4 (9.7)	9.7 (9.9)	10.1 (10.3)

The first and the last introns may reside in untranslated or coding regions. Sizes are expressed in nucleotides (nt). Estimates of 5′ss and 3′ss strength are calculated after excluding introns smaller than 32 nt (see *Materials and Methods*).

It has also been reported that the splicing-enhancing effects of the CBC may be distance dependent (Qiu *et al.* 2007). In investigating this hypothesis, we uncovered a significant correlation between the strength of the first 5′ss and its distance from the CBC (6967 observations; Kendall’s tau = 0.16, *P* < 0.001) (Figure 1B). The robustness of this positive relationship is upset when the median first exon size ranges between ∼180 and 250 nt (1243 observations)—during this interval the progressive increase of the strength of the first 5′ss stops (Figure 1B)—whereas it is virtually unaffected by the size of the first intron (6967 observations; Kendall’s tau partial = 0.15, *P* < 0.001). In contrast, the strength of 5′ss and the size of the upstream exon are not correlated in the gene body (19,504 observations; Kendall’s tau partial = ∼0, *P* = 0.65), and they are only weakly correlated at the gene 3′ end (7107 observations; Kendall’s tau partial = 0.04, *P* < 0.001). These results suggest that the putative splicing-enhancing effects of the CBC in *Drosophila* might vanish with distance. Our observations hint at a maximum interval over which the putative splicing-enhancing effects of the CBC might extend (*i.e.*, ∼250 nt). Beyond this distance, both the strength of first 5′ss and first exon length are tightly and positively coupled with first intron size.

### The splicing-enhancing properties of the CBC and the suppressive effects of U1 on cleavage/polyadenylation might shape intron-exon size at the gene 5′ end

Experimental evidence demonstrates that U1 plays a critical role in safeguarding the integrity of the eukaryotic transcriptome. In *Drosophila*, as well as human and mouse, 5′ss-bound U1 counteracts premature termination by cleavage/polyadenylation at the nearby PAS, a process known as telescripting (Berg *et al.* 2012). If the distance-dependent suppressive effects of 5′ss-bound U1 are critical for the generation of intact functional transcripts, then we should observe a nonrandom distribution in the size of the interval between two subsequent donor splice sites—the size of an intron plus its next exon (hereafter, IpE unit). Rather, the size of IpE units may approximate the median distance over which the efficiency of the protective action of U1 is optimal [*i.e.*, ∼500 nt in *D. melanogaster* (Berg *et al.* 2012)].

**Figure 2:**
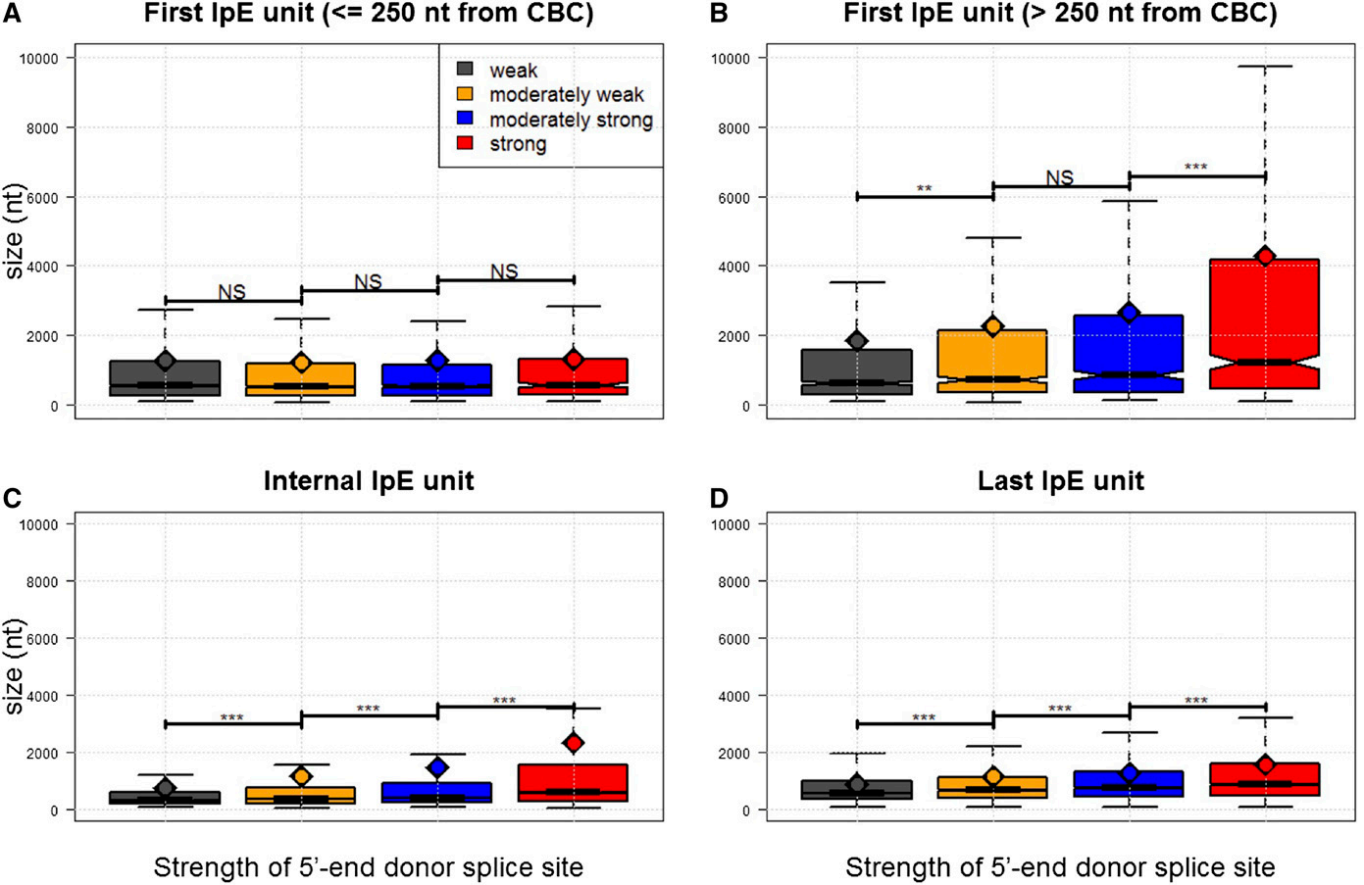
Relationship between the size of intron plus its next exon (IpE) units in nucleotides (nt), and the strength of the associated 5′-end 5′ss, according to intragenic position: cap-proximal first IpE units (A), cap-distal first IpE units (B), internal IpE units (C), and last IpE units (D). First IpE units are separated depending on their distance from the CBC (*i.e.*, ≤ 250 nt and > 250 nt), and the strength of the associated 5′ss is divided in quartiles (weak ≤ 7.7 < moderately weak ≤ 9.6 < moderately strong ≤ 11.0 < strong). Median and mean strength values are illustrated with horizontal black bars and full diamonds, respectively. NS and asterisks over the bars describe the significance (or lack thereof) of statistical differences. * *P* < 0.05, ** *P* < 0.01, *** *P* < 0.001.

**Figure 3:**
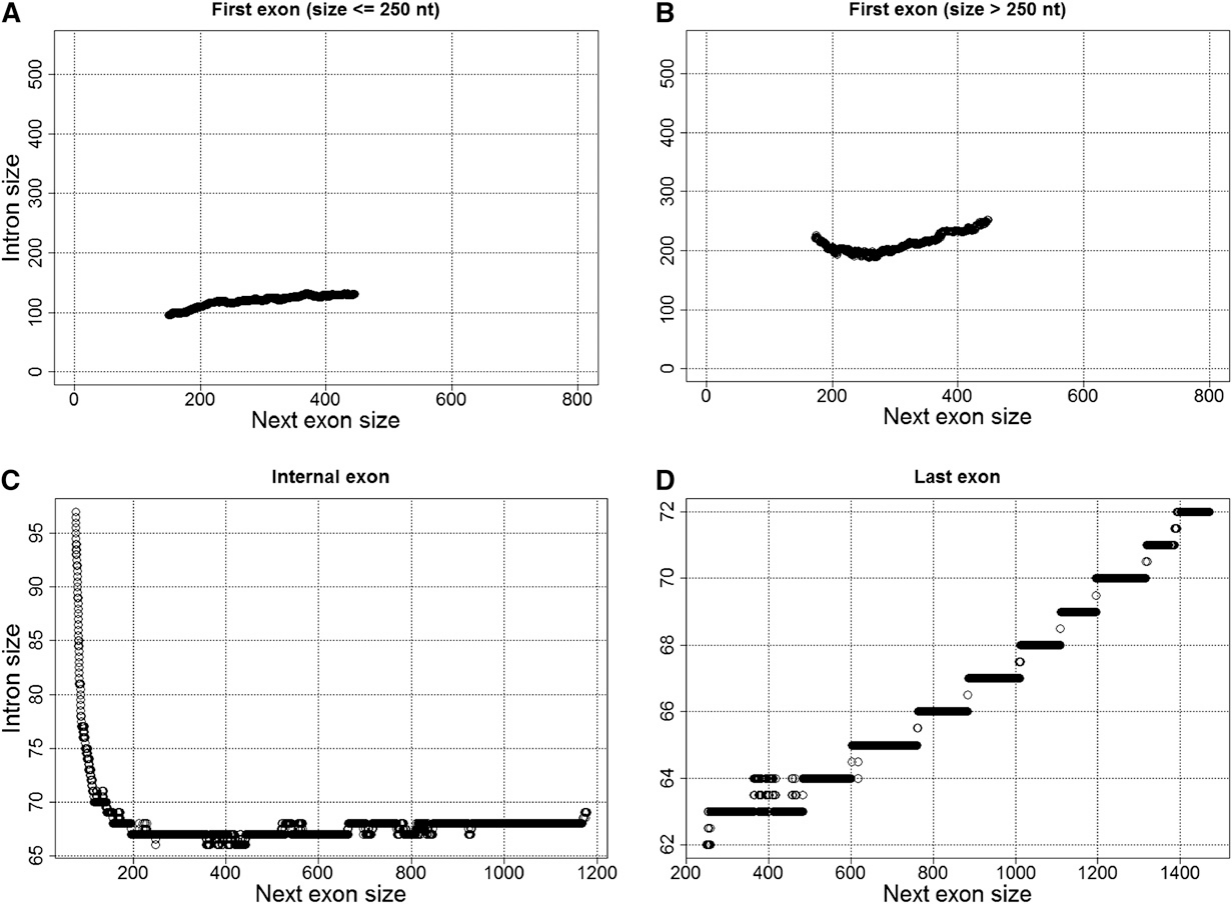
Relationship between the sizes of first cap-proximal and cap-distal introns (A), (B), internal introns (C), and last introns (D) and their next exon. Data were ranked according to the *x*-axis variable, and subsequent medians of 2000 observations (step size of 1) for *x*- and *y*-axis variables were estimated and plotted.

To search for putative signatures of telescripting at the 5′ ends of genes, we studied 1) the size of first IpE units, and 2) the relationship between the size of introns to their next exon within these units. We examined first IpE units whose 5′ end donor splice site resides either within or outside the 5′-most 250 nt separately (we termed them cap-proximal and cap-distal IpE units, respectively). This partition might help uncover putative interactions between the processes of capping and telescripting. We found that the size of cap-proximal IpE units is uniform (median: 553 nt) irrespective of the strength of the upstream 5′ss (Kruskal-Wallis test, *P* = 0.72) (Figure 2A). In contrast, the size of cap-distal IpE units increases with the strength of the 5′ss (Kruskal-Wallis test, *P* < 0.001) (Figure 2B). Furthermore, whereas the sizes of cap-proximal introns and their next exon are weakly positively correlated (3748 observations; Kendall’s tau = 0.04, *P* < 0.001) (Figure 3A), the relationship between the sizes of cap-distal introns and their next exon exhibits a ∪-shaped pattern (Figure 3B). More specifically, the size of first cap-distal introns 1) decreases steeply while second exons expand to up to ∼200 nt in size (1239 observations; Kendall’s tau = –0.08, *P* < 0.001), and 2) weakly increases when second exons are larger than 200 nt (1980 observations; Kendall’s tau = 0.03, *P* < 0.005). These observations demonstrate that intron-exon structure may differ dramatically at the gene 5′ end. They reveal constraints that fall within the action range of telescripting in *Drosophila*, *i.e.*, the sizes of introns and their next exon covary such that the median length of the IpE unit is ≤ ∼500 nt (Berg *et al.* 2012).

Under a model where selection pressure keeps two subsequent 5′ss at a distance that minimizes the risk of premature cleavage/polyadenylation, the CBC-enhanced recruitment of U1 should produce two effects: 1) it should compensate for the weak strength of cap-proximal splice sites, and 2) it should maximize the protective effects of telescripting at the gene 5′ end. Under this same model, large (>> 500 nt) IpE units are at major risk of premature cleavage/polyadenylation, unless additional protective features come into play (see below).

### Telescripting and the nonrandom internal structure of Drosophila protein-coding genes

Putative telescripting-associated constraints on IpE unit size are also detectable in internal intragenic regions. Namely, the size of internal introns, and that of their next exon, are negatively correlated within the protective range of 5′ss-bound U1s (*e.g.*, when exon size is ≤ 400 nt: 14,428 observations; Kendall’s tau = –0.07, *P* < 0.001), whereas they are positively, albeit very weakly, correlated beyond this range (*e.g.*, when exon size > 500 nt: 3900 observations; Kendall’s tau = 0.01, *P* = 0.03) (Figure 3C). Additionally, internal IpE units in *Drosophila* genes are significantly smaller than peripheral units [medians: 417 nt *vs.* 640 nt (5′-end) and 735 nt (3′-end), Kruskal-Wallis test, *P* < 0.001]. Finally, the size distribution of internal IpE units differs significantly from a set of distributions generated by randomly sampling introns and exons from our dataset (*P* < 0.001) (Figure S1). These observations suggest that there are constraints on the internal intron-exon structure of protein-coding genes in *D. melanogaster* that approximate the action range of telescripting in this species (Berg *et al.* 2012).

### Using the degree of DNA strand asymmetry of polyadenylation motifs as a tool to detect and measure the efficiency of telescripting effects

The median size of internal IpE units increases alongside the strength of the 5′-end 5′ss, ranging from 294 nt (weak 5′ss), through 328 nt (weak to moderate) and 374 nt (moderate to strong), to 513 nt (strong 5′ss) (Figure 2C). This trend resembles what was observed for first cap-distal IpE units (Figure 2B), and it largely reflects the increasing length of introns in these IpE units. That noted, the 5′ss strength-associated lengthening of IpE units might also indicate that the action range of telescripting increases along with the strength with which U1 binds the 5′ss. We tested this hypothesis leveraging the degree of DNA strand asymmetry (DSA) of the polyadenylation AATAAA motif. A sequence motif’s negative (positive) DSA is likely to reflect selection against (in favor of) that motif (Mitchell and Bridge 2006; Farlow *et al.* 2012). Because cryptic PAS may represent a risk for the generation of intact mRNAs (Kaida *et al.* 2010; Berg *et al.* 2012), we hypothesized that the polyadenylation AATAAA motif is generally selected against in preterminal regions, unless they are adequately shielded by nearby 5′ss-bound U1s.

**Figure 4:**
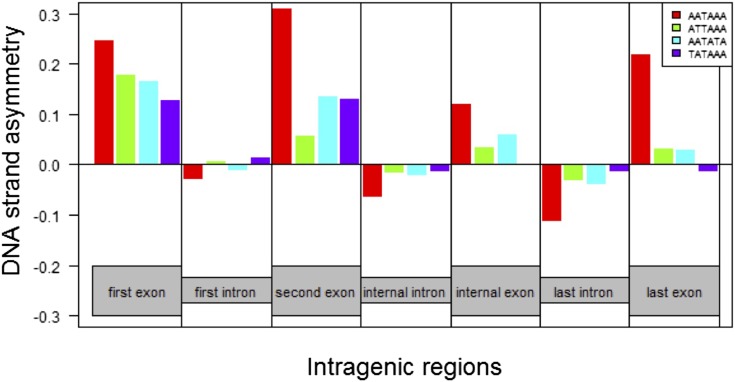
DNA strand asymmetry (DSA) of the canonical (strong) polyadenylation motif AATAAA, and putative polyadenylation motifs ATTAAA, AATATA, and TATAAA. DSA values are estimated for (first, second, internal, and last) exons and (first, internal, and last) introns separately.

We found that the average DSA of AATAAA within *Drosophila* introns is indeed negative (–0.06) (Figure 4). In introns, DSA_AATAAA_ is ∼3-, ∼5-, and 15-fold more negative compared to that of three putatively weaker polyadenylation motifs (DSA_ATTAAA_, DSA_AATATA_, and DSA_TATAAA_, respectively), and ∼4-fold more negative compared to the DSA value of two AATAAA anagrams, TAAAAA, and AAAAAT (data not shown). The average DSA_AATAAA_ is also > 3-fold more negative at the gene 3′ end than at the 5′ end (DSA_First introns_: –0.03; DSA_Internal introns_: –0.07, DSA_Last introns_: –0.11). Finally, levels of counter-selection of intronic AATAAA decrease (*i.e.*, DSA_AATAAA_ is relatively less negative) when the strength of the upstream 5′ss increases (Table S2). These patterned variations suggest that the risk of premature polyadenylation and/or the efficiency of telescripting might differ between the gene 5′ end and 3′ end, and that the strength of the 5′ss may indeed bear on the action range of telescripting.

We also examined the degree of DNA strand asymmetry of AATAAA in exons (Figure 4). AATAAA is particularly favored not only in last exons (DSA_AATAAA_ = 0.21)—where it may serve to regulate gene expression (Beaudoing *et al.* 2000; Yang *et al.* 2009)—but also in first and second exons (DSA_AATAAA_ = 0.24 and 0.30, respectively). As rationalized above, the excess of cryptic PAS at the gene 5′ end may reflect efficient U1-mediated silencing of polyadenylation in this region. Elegant work by Andersen *et al.* (2012) and Guo *et al.* (2011) provides some support for this claim, demonstrating that functional polyadenylation motifs at the gene 5′ end are silenced by sufficiently close 5′ss-bound U1.

Finally, we detected a positive relationship between DSA_AATAAA_ in first or second exons and the size of the intervening intron (Figure S2, A and B). This observation provides some ground to speculate on why shielded PAS at the gene 5′ end might be favored (see *Discussion*).

### Coordinated regulation of splicing and 3′-end pre-mRNA processing

Splicing can be problematic for accurate 3′ end formation in that the suppressive effects of 5′ss-bound U1s might perturb the accurate processing of downstream cleavage and polyadenylation signals, particularly when the U1-bound 5′ss is very close to the polyadenylation site (van Gelder *et al.* 1993; Gunderson *et al.* 1994). It follows that intron-exon structure at the gene 3′ end should exhibit characteristics that permit the coexistence of 3′-most 5′ss and downstream 3′ end processing signals.

In testing this hypothesis, we detected a significant and positive relationship between the size of last intron and that of the last exon (7130 observations; Kendall’s tau = 0.16, *P* < 0.001; Figure 3D). We also verified that last exons are quite large, 848 nt (596 nt) on average (median), that is > 2-fold larger than the first and internal exons (Hong *et al.* 2006; Kruskal-Wallis test, *P* < 0.001; Table 1). These observations suggest that the size of 3′ end IpE units is free from upper-bound constraints, unlike the size of many first and internal IpE units (Figure 3, B and C).

Furthermore, we found that the strength of the last 5′ss is significantly and positively correlated with the size of the last exon (after correcting for last intron size) (7130 observations; Kendall’s tau = 0.09 (0.07), *P* < 0.001). Nowhere else along the gene do we detect a significant correlation between exon size and upstream 5′ss strength (Kendall’s tau = ∼0; *P* = 0.15). This indicates that increasing exon length significantly contributes to the positive coupling between the size of last (but not first or internal) IpE units and the strength of the 5′-end 5′ss (Figure 2D). This observation is compatible with a model where telescripting shapes gene structure in *Drosophila*. If the efficiency of telescripting truly increases alongside the relative strength of the 5′ss (as proposed above), then, in order for accurate mRNA 3′ end processing to take place, progressively stronger last-intron 5′ss should be coupled with increasingly large next exons.

Finally, we found that, on average, last introns have stronger 3′ss compared to first and internal introns (Kruskal-Wallis test, *P* < 0.001). This observation is surprising given the exceedingly small size of last introns in *Drosophila* (Hong *et al.* 2006; Table 1). This suggests that splicing might be disfavored at the pre-mRNA 3′ end, such that only introns with sufficiently good splice sites can reside at this location.

### Cryptic donor splice site and polyadenylation motifs co-occur preferentially in D. melanogaster large first and internal introns

The findings described above show that a considerable number of IpE units at the gene 5′ end, and in the gene body, have a size that exceeds the action range of telescripting in *D. melanogaster*. This condition is potentially deleterious because it eases undesired premature termination by cleavage/polyadenylation. However, U1-recruiting cryptic donor splice sites might help to prevent the processing of otherwise unprotected cryptic PAS in large IpE units. To test this hypothesis, we investigated the distribution of the 5′ss-related GGTAAG motif as well as the co-occurrence of GGTAAG and the polyadenylation motif AATAAA within *Drosophila* genes.

We found that the 5′ss-related GGTAAG motif is counter-selected in introns and exons (average DSA_GGTAAG_ = –0.19 and –0.27, respectively), in line with previous findings (Farlow *et al.* 2012), and that this counter-selection is more relaxed in introns with strong 5′ end 5′ss compared to introns with weak 5′ end 5′ss (Table S2). This negative selection notwithstanding, about 4% (*n* = 1417) of the surveyed introns (and 2.5% of exons; *n* = 1069) contain at least one GGTAAG, and the motifs GGTAAG and AATAAA co-occur more frequently 1) in > 500-nt introns compared to smaller introns [18% *vs.* 0.07%, respectively], and 2) downstream from, rather than within, the first 500 nt, in ∼1000-nt introns (96% *vs.* 4%, respectively; χ^2^ = 934.6, *P*-value < 0.001). Finally, we observed that the AATAAA motif resides preferentially toward the intron 3′ end, downstream (rather than upstream) from GGTAAG (χ^2^ = 69.49, *P*-value < 0.001; Figure S3 and Figure S4). Taken together, these findings lend some support to the hypothesis that U1 binding to cryptic 5′ss in *Drosophila*’s large introns may help suppress cleavage/polyadenylation at otherwise unshielded cryptic PAS.

### Conservation between Drosophila species

To assess the validity of our study, we replicated most of our analyses on the annotated genome of *D. yakuba* (release FB2015_01; ftp://ftp.flybase.net/genomes/Drosophila_yakuba/dyak_r1.04_FB2015_01/fasta/dyak-all-chromosome-r1.04.fasta.gz; ftp://ftp.flybase.net/genomes/Drosophila_yakuba/dyak_r1.04_FB2015_01/gtf/dyak-all-r1.04.gtf.gz), a distant relative of *D. melanogaster* (∼10 million yr of sequence divergence). These analyses corroborate virtually all of the relationships and trends that we document for *D. melanogaster* (a detailed description is provided in Table S3, Table S4, Table S5, Figure S5, Figure S6, Figure S7, Figure S8, and Figure S9). Two discrepancies, which we detected, do not seem to have significant consequences for our overall conclusions. First, we found no significant negative association between the size of cap-proximal first introns and the 5′ most 5′ss for *D. yakuba* (within the first 200 nt from the CBC: 2937 observations; Kendall’s tau = –0.001, *P* = 0.89). Second, for *D. yakuba* the size of cap-proximal IpE units may vary depending on the strength of the upstream 5′ss (rather than being uniform as is for *D. melanogaster*). The magnitude of this variation in size is marginal however (Kruskal-Wallis test, *P* = 0.02).

## Discussion

**Figure 5:**
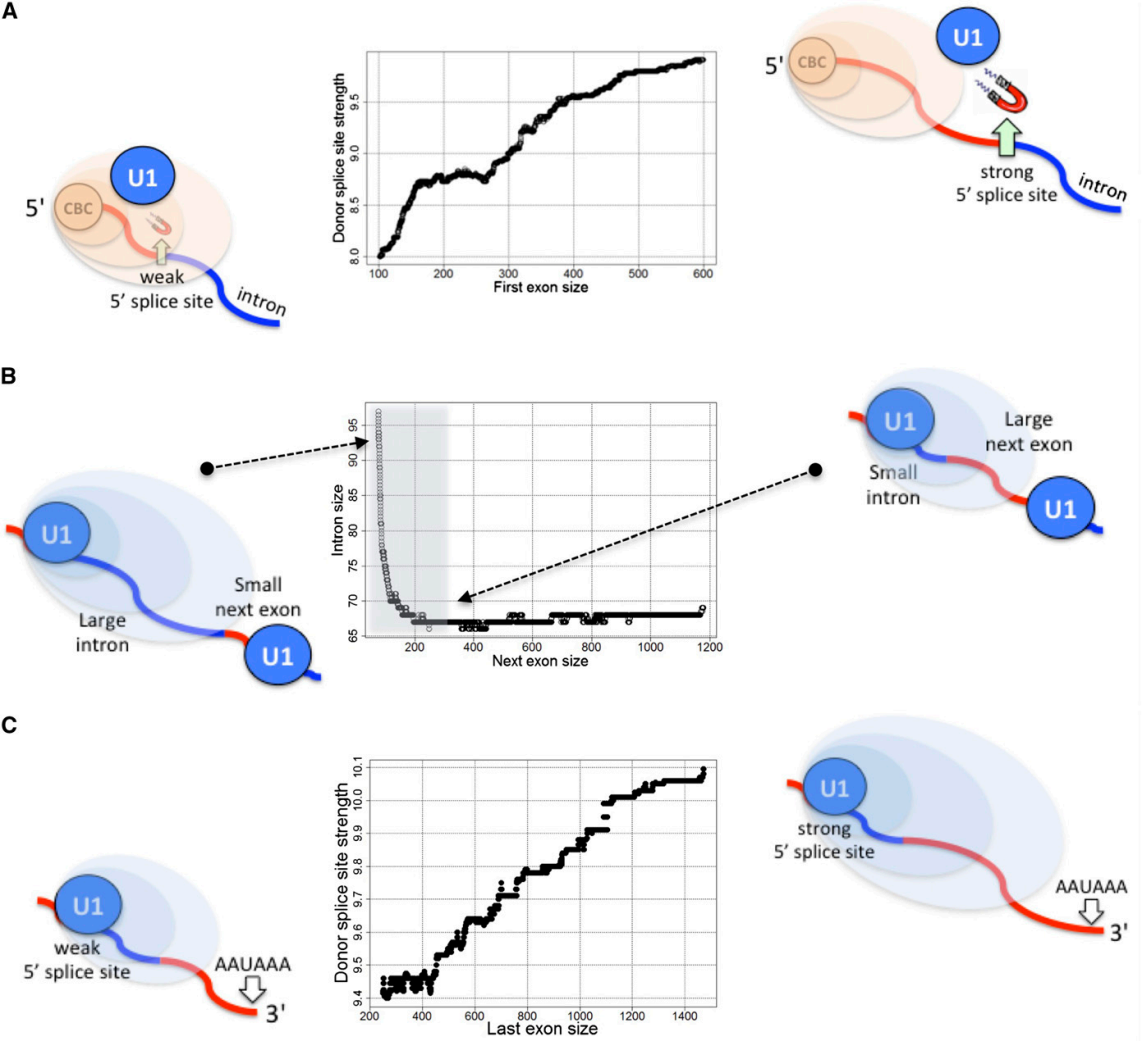
Principal conclusions of this study. (A) Because the cap binding complex (CBC) enhances splicing at the 5’ end of pre-mRNAs, introns nearby the CBC may not need to evolve (or maintain) a strong donor splice site. (B) The action range of telescripting may impose constraints on the distance between two subsequent donor splice sites so as to minimize the risk of premature cleavage/polyadenylation within that interval. (C) The strength of the last 5′ss is positively correlated with the size of the last exon (after correcting for last intron size) such that telescripting effects do not suppress the process of 3′ end formation.

We have presented several findings which suggest that interacting mRNA-associated processes, including capping, splicing, cleavage/polyadenylation, and telescripting might play a role in molding the structural properties of protein-coding genes in *Drosophila*. The principal conclusions of this study are summarized in Figure 5.

We have detected size constraints in the internal regions and at the 5′ end of *Drosophila* genes that, in theory, maximize telescripting effects, thus minimizing the risk of premature cleavage/polyadenylation in nascent transcripts. At the gene 3′ end, on the other hand, our observations suggest that telescripting effects are often circumvented, so that the process of 3′ end formation is facilitated.

Our analyses further indicate that, as IpE unit size increases with 5′ss strength, so may the efficiency of telescripting. More specifically, we found that counter-selection of the AATAAA motif (a target of cleavage/polyadenylation factors) is more pronounced in IpE units with weak 5′-end 5′ss compared to IpE units with strong 5′-end 5′ss. This observation may indicate that strong 5′ss protect pre-mRNAs from premature termination more efficiently than weak 5′ss. In line with this scenario, introns with a weak 5′ss are more likely to undergo alternative cleavage/polyadenylation compared to introns with a strong 5′ss (Tian *et al.* 2007; Wu *et al.* 2011). The relationship between donor splice site strength and intronic DSA_AATAAA_ might also indicate that intron-bound cleavage/polyadenylation factors perturb splicing, *e.g.*, intron-bound cleavage/polyadenylation factors might disturb the recruitment of U1 at the upstream canonical donor splice site. If so, then weakly counter-selected intronic polyA sites would preferentially occur downstream of strong (rather than weak) donor splice sites, which facilitate the recruitment of U1. This scenario is consistent with previously proposed models that invoke competing splice and cleavage and polyadenylation reactions (Catania and Lynch 2008, 2013; Martinson 2011).

Furthermore, we have found that the 5′ss-like motif GGTAAG and the polyadenylation motif AATAAA co-occur along introns more frequently outside of the protective range of authentic 5′ss-bound U1s than within. We propose that this nonrandom association/distribution of cryptic donor splice sites might help suppress polyadenylation at cryptic PAS that are insufficiently proximal to authentic U1-bound 5′ss.

In addition, we have detected a signature of selection that favors the AATAAA motif not only in last exons, where this motif plays a role in the process of mRNA 3′-end formation (Proudfoot 1991), and helps regulate gene expression (Beaudoing *et al.* 2000; Yang *et al.* 2009), but also in first exons and second exons. The over-representation of the AATAAA hexamer at the 5′ end of *Drosophila*’s genes is in line with previous experimental observations (Guo *et al.* 2011), and lends further support to the notion that telescripting operates at this location to suppress premature polyadenylation (Andersen *et al.* 2012). The reasons why (silenced) cryptic PAS should be favored at the gene 5′ end are still unclear. Drawing from our previous theoretical work (Catania and Lynch 2013), we speculate that exonic AATAAAs may act as splicing enhancer. In particular, exonic AATAAAs might serve to reroute and advantageously sequester cleavage/polyadenylation factors that could otherwise access and perturb the splicing of the neighboring intron. Under this model, the relative excess of AATAAA in the first and second exons of *Drosophila*’s genes might aid the splicing of first introns, which in turn exhibit weaker levels of selection against cryptic PAS compared to downstream introns. Consistent with this model, and with the notion that large introns are more prone to inefficient cotranscriptional splicing than small introns (Khodor *et al.* 2011), we also found that the DNA strand asymmetry of AATAAA in first and second exons increases alongside first intron size. If exonic PAS help the splicing of neighboring introns, this effect might take place elsewhere along the gene. It is less surprising then that we detected a relative excess of AATAAA in internal exons that are downstream of strong 5′ss, which are typically associated with large introns. This reasoning may even be extended to last-exon AATAAAs, which would help splicing of last introns by reducing the risk that unengaged cleavage/polyadenylation factors access cryptic PAS in these introns.

Our analyses reveal significant variations in the average splice site strength along the genes of *D. melanogaster*. At face value, these variations are counterintuitive: splice sites are the weakest at the gene 5′ end (where introns are the largest, on average), and rather strong at the gene 3′ end (where introns are the smallest, on average). It follows that, whereas splice site strength and intron size are overall positively correlated within a certain intragenic location, across intragenic regions this relationship may disappear (*e.g.*, small introns at the pre-mRNA tail have relatively strong splice sites). We interpret this pattern of longitudinal decoupling between intron size and splice site strength as indicating that splicing in *Drosophila* is assisted at the pre-mRNA 5′ end but not at its 3′ end.

Alongside previous findings for human and yeast (Colot *et al.* 1996; Lewis *et al.* 1996), our observations suggest that the CBC may play a splicing-enhancing role at the pre-mRNA 5′ end—a hypothesis that requires experimental verification. An alternative interpretation of the variations in splice site strength that we detected along *Drosophila* genes is that splicing efficiency differs among introns, and that first introns are more prone to be inefficiently spliced, at least in part because of their relatively weak 5′ splice sites. This view provides an additional explanation for why first introns in *Drosophila* are retained more frequently than others (Khodor *et al.* 2012). Yet, it does not seem to justify why short first introns, which we document as displaying the weakest 5′ss on average, have a lower (rather than higher) frequency of retention compared to that of larger first introns (Khodor *et al.* 2012). Although intron retention can reflect or be viewed as inefficient splicing, it might also be regulatory (Braunschweig *et al.* 2014). We propose that the first intron retention events documented by Khodor *et al.* (2012) may also reflect a regulated delay. The retention of first introns throughout transcription would prolong the sojourn time of 5′ss-bound U1 at the pre-mRNA 5′ end, thereby permitting U1 to advantageously suppress premature polyadenylation at PAS that are selectively favored in first and second exons. This delayed processing of first introns is compatible with our proposition that first intron splicing may be assisted in *Drosophila*, and allows the findings of Khodor *et al.* (Khodor *et al.* 2011, 2012) to be integrated with previously documented telescripting effects at the gene 5′ end (Guo *et al.* 2011; Andersen *et al.* 2012).

We further propose that splicing may be disfavored at the pre-mRNA 3′ end. This proposition is in accord with previous experimental findings in yeast (Tardiff *et al.* 2006), with expectations given the scarcity of introns in eukaryotic 3′ UTRs (Hong *et al.* 2006), and with previous theoretical work (Catania and Lynch 2008, 2013). Our empirical observations, *i.e.*, that changes in last intron and last exon size are related and may have unequal magnitude, raise several questions, one among them being “how do minor differences in the last intron length mechanistically translate into differences of hundreds of nucleotides in the last exon?” While leaving this and other questions unanswered, our observations offer potential insights into the evolution of gene tail structure. We speculate that selection for a relatively large expansion of last exon size may be promoted by a minor increase in the strength of the last donor splice site (which may or may not be followed by some minor intron expansion). This selection for last exon expansion would have the beneficial effects of keeping the 3′ termination signals and the upstream 5′ss-bound U1 separated by a distance that is sufficiently large to circumvent telescripting effects and, thus, to facilitate both cotranscriptional splicing and mRNA 3′-end formation. Should telescripting effects not be adequately circumvented, splicing at the mRNA tail could still be completed post-transcriptionally, which is consistent with the observed excess of post-transcriptional splicing at the mRNA 3′-end compared to the upstream regions (Khodor *et al.* 2012).

Finally, we are aware that the high degree of interrelatedness between many (if not all) of the variables that we have investigated in this study (intron size, splice site strength, DSA_AATAAA_, etc.) complicates attempts to draw conclusions concerning underlying molecular dynamics, and might yield erroneous interpretations. In consequence, we cannot definitively rule out the possibility that some of the evidence presented in support of the proposed role of mRNA-associated processes in molding gene structure may be inaccurate. With this caveat in mind, we attempted to eliminate false signals whenever possible, using partial correlations or control sequence motifs. Not only do our conclusions withstand this scrutiny, they are also sustained by the coherence of the detected patterns. These patterns are in accord with U1-dependent definition (Catania and Lynch 2013)—a model that puts forward a mechanistic explanation for trends of eukaryotic gene architecture and expression that uniquely speaks to recent experimental results, which are poorly accounted for by previous models.

### Conclusions

The features and the relationships that we document support the idea that intracellular processes in conjunction with population-genetic forces may shape gene structure in *Drosophila*. They support well-established traits of pre-mRNA-associated processes (*e.g.*, the splicing-enhancing effects of the CBC), and potentially extend the repertoire of these traits with novel hypotheses that can be verified experimentally. In one example, the degree of DNA strand asymmetry of cryptic PAS motifs might serve as a simple but powerful tool to detect and measure *in silico* the efficiency of telescripting effects within the intragenic regions of eukaryotic species.

The extent to which the proposed effects of mRNA-associated processes in *Drosophila* may extend to other eukaryotes is an open question that warrants further investigation. Many eukaryotic species, such as humans for example, have considerably longer introns compared to *Drosophila* and there is compelling experimental evidence that in human and mouse U1 telescripting effects extend for ∼1000 nt (rather than 500 nt as in *D. melanogaster*). Human and mouse are therefore excellent candidate systems to examine. These investigations would also furnish a test of the validity and the generalizability of the patterns observed for *Drosophila*.

We anticipate that much will be gained from examining the features that distinguish fruit flies from other species. Between-species differences in gene structure might flag different properties of the intracellular environment (*e.g.*, distinct levels and types of DNA methylation (Gelfman *et al.* 2013; Zhang *et al.* 2015), differences in the strength/effectiveness of natural selection (Lynch 2007), and/or distinct degrees of compensatory relationships between the intracellular and the population-genetic environment (this study). This information should help further our understanding of the relative contribution of selection and intracellular forces to the evolution of eukaryotic genes.

## Supplementary Material

Supplemental Material
