# Supplementary material for: mRNA-Associated Processes and Their Influence on Exon-Intron Structure in Drosophila melanogaster
Source: G3 (Bethesda). 2016 Mar 28;6(6):1617–26. doi: 10.1534/g3.116.029231 (PMC4889658; doi:10.1534/g3.116.029231)
Supplement: Supplemental Material [file supp_6_6_1617__index.html]

Supplemental Material 

# mRNA-Associated Processes and Their Influence on Exon-Intron Structure in *Drosophila melanogaster*

Supplemental Material for Lepennetier and Catania, 2016

Supplemental Material

**Files in this Data Supplement:**

- Figure S1 - Comparison of actual Intron plus next Exon (IpE) unit size distribution with distributions generated by random sampling of introns and exons from the studied dataset (see Materials and Methods). (.pdf, 243 KB)
- Figure S2 - Relationship between the sizes of first introns and the degree of DNA strand asymmetry (DSA) for the polyadenylation AATAAA motif in (A) first exons and (B) second exons. (.pdf, 303 KB)
- Figure S3 - Spatial distribution of the polyadenylation AATAAA motif and the donor splice site-like GGTAAG motif along (the sense DNA strand of) *D. melanogaster* small (≤500 nt) and large (>500 nt) introns. (.pdf, 198 KB)
- Figure S4 - Spatial distribution of the polyadenylation AATAAA motif and the donor splice site-like GGTAAG motif along (the sense and the antisense DNA strand of) all of the *D. melanogaster introns*. (.pdf, 204 KB)
- Figure S5 - Relationship between first exon size (in nucleotides) and (A) first intron size or (B) strength of first-intron donor splice site. (.pdf, 254 KB)
- Figure S6 - Relationship between the size of an Intron plus its next Exon (IpE) units in nucleotides (nt) and the strength of the associated 5'-end 5'ss, according to intragenic position. (.pdf, 383 KB)
- Figure S7 - Relationship between the sizes of first cap-proximal and capdistal introns (A, B), internal introns (C), and last introns (D) and their next exon. (.pdf, 373 KB)
- Figure S8 - DNA strand asymmetry (DSA) of the canonical (strong) polyadenylation motif AATAAA and the putative polyadenylation motifs ATTAAA, AATATA, and TATAAA. DSA values are estimated separately for (first, second, internal, and last) exons and (first, internal, and last) introns. (.pdf, 89 KB)
- Figure S9 - Relationship between the log-transformed sizes of first introns and the degree of DNA strand asymmetry (DSA) for the polyadenylation AATAAA motif in (A) first exons and (B) second exons. (.pdf, 240 KB)
- Table S1 - Kendall's tau correlation coefficients describing the strength of the association between the sizes of first, internal, or last introns and the quality of their corresponding 5'ss or 3'ss. (.pdf, 74 KB)
- Table S2 - Degree of DNA strand asymmetry (DSA) of the polyadenylation AATAAA motif and the donor splice site-like GGTAAG motif in *D. melanogaster*'s (internal and peripheral) introns with weak (≤7.7; ≤8.1) and strong (>11.0; >11.7) 5'ss and 3'ss respectively. (.pdf, 88 KB)
- Table S3 - Summary statistics on the surveyed *D. yakuba* introns partitioned according to their relative intragenic position. (.pdf, 82 KB)
- Table S4 - Kendall's tau correlation coefficients that describe the strength of the association between the sizes of first, internal, or last introns and the quality of their corresponding 5'ss or 3'ss. (.pdf, 82 KB)
- Table S5 - Degree of DNA strand asymmetry (DSA) of the polyadenylation AATAAA motif and the donor splice site-like GGTAAG motif in *D. melanogaster*'s (internal and peripheral) introns with weak (≤7.7; ≤8.0) and strong (>11.0; >11.6) 5'ss and 3'ss respectively. (.pdf, 85 KB)
