# Supplementary material for: mRNA-Associated Processes and Their Influence on Exon-Intron Structure in Drosophila melanogaster
Source: G3 (Bethesda). 2016 Mar 28;6(6):1617–26. doi: 10.1534/g3.116.029231 (PMC4889658; doi:10.1534/g3.116.029231)
Supplement: Supplemental Material [file supp_g3.116.029231_FigureS1.pdf]

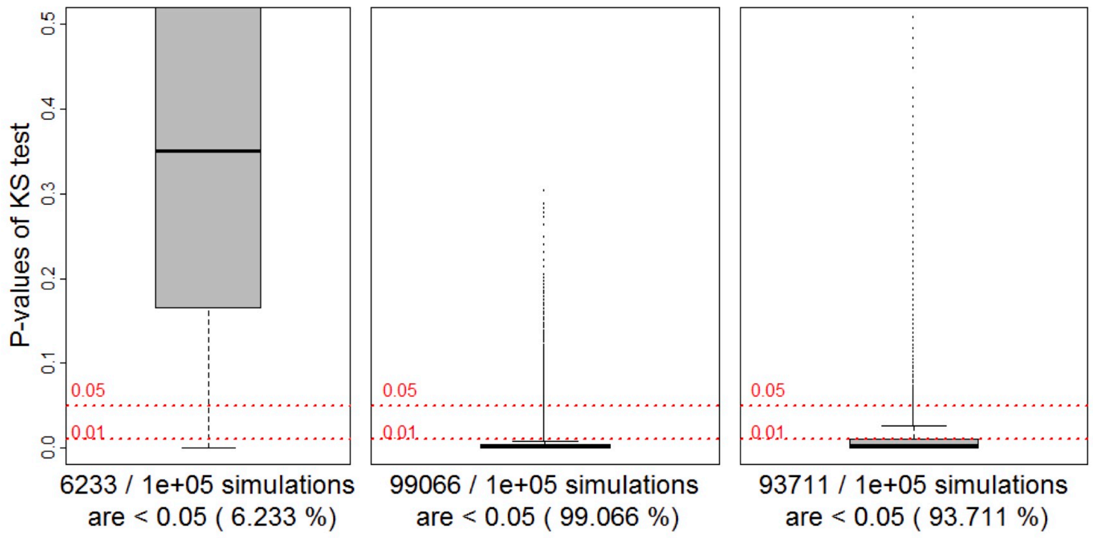


**Fig. S1.** Comparison of actual Intron plus next Exon (IpE) unit size distribution with distributions generated by random sampling of introns and exons from the studied dataset (see Materials and Methods). The boxplots illustrate the distribution of the *P*-values (relative to the Kolmogorov-Smirnov (KS) test) estimated for each of 100,000 simulations. The horizontal red dashed lines indicate the alpha levels of 0.05 and 0.01. The final *P*-value was estimated as N/100,000, *i.e.*, the number of times that the test was non-significant ($P$-value $< 0.05$) divided by the number of simulations.
