# Supplementary material for: mRNA-Associated Processes and Their Influence on Exon-Intron Structure in Drosophila melanogaster
Source: G3 (Bethesda). 2016 Mar 28;6(6):1617–26. doi: 10.1534/g3.116.029231 (PMC4889658; doi:10.1534/g3.116.029231)
Supplement: Supplemental Material [file supp_g3.116.029231_FigureS2.pdf]

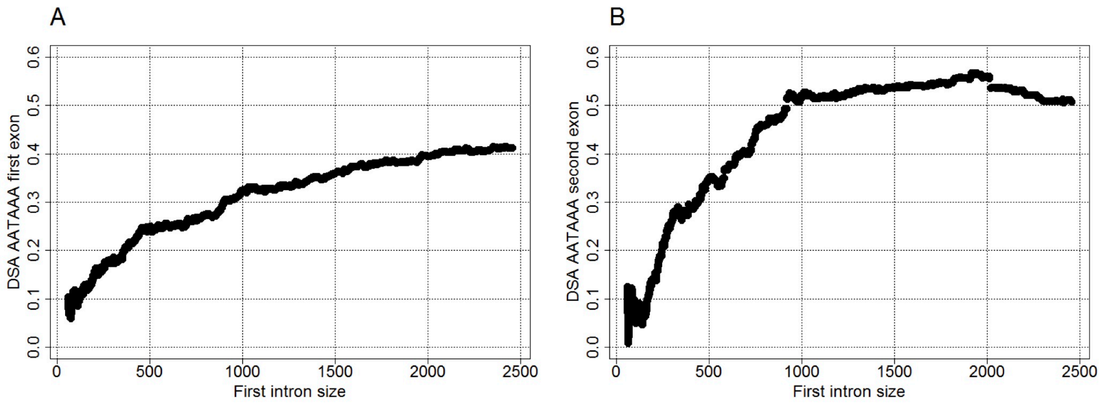

Fig. S2. Relationship between the sizes of first introns and the degree of DNA strand asymmetry (DSA) for the polyadenylation AATAAA motif in (A) first exons and (B) second exons. Data were ranked according to the X-axis variable. Subsequent medians of 2,000 observations (step size of 1) for X- and Y-axis variables were estimated and plotted.
