# Supplementary material for: mRNA-Associated Processes and Their Influence on Exon-Intron Structure in Drosophila melanogaster
Source: G3 (Bethesda). 2016 Mar 28;6(6):1617–26. doi: 10.1534/g3.116.029231 (PMC4889658; doi:10.1534/g3.116.029231)
Supplement: Supplemental Material [file supp_g3.116.029231_FigureS4.pdf]

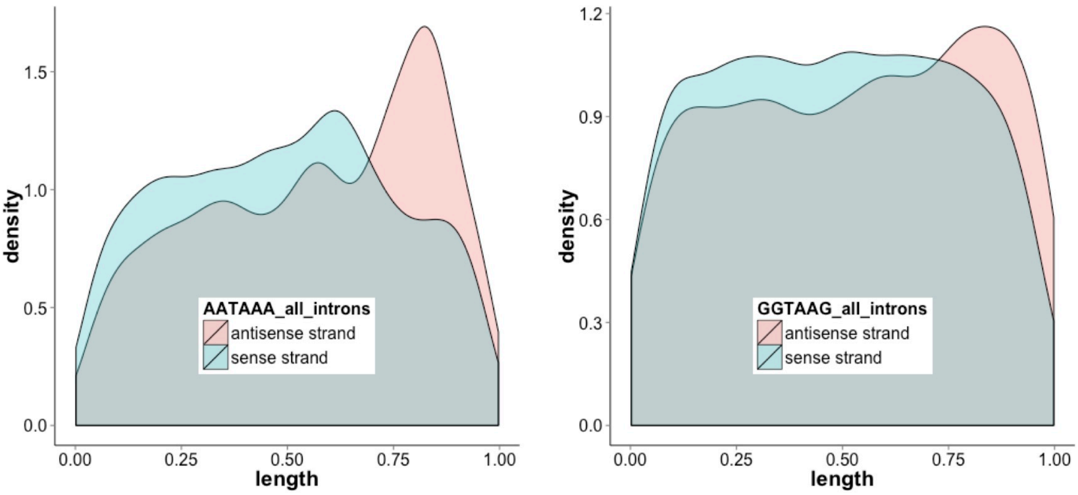

Fig. S4. Spatial distribution of the polyadenylation AATAAA motif and the donor splice site-like GGTAAG motif along (the sense and the antisense DNA strand of) all of the *D. melanogaster* introns. The x-axis shows the ratio between motif position (from intron start) and intron length.
