# Supplementary material for: mRNA-Associated Processes and Their Influence on Exon-Intron Structure in Drosophila melanogaster
Source: G3 (Bethesda). 2016 Mar 28;6(6):1617–26. doi: 10.1534/g3.116.029231 (PMC4889658; doi:10.1534/g3.116.029231)
Supplement: Supplemental Material [file supp_g3.116.029231_FigureS5.pdf]

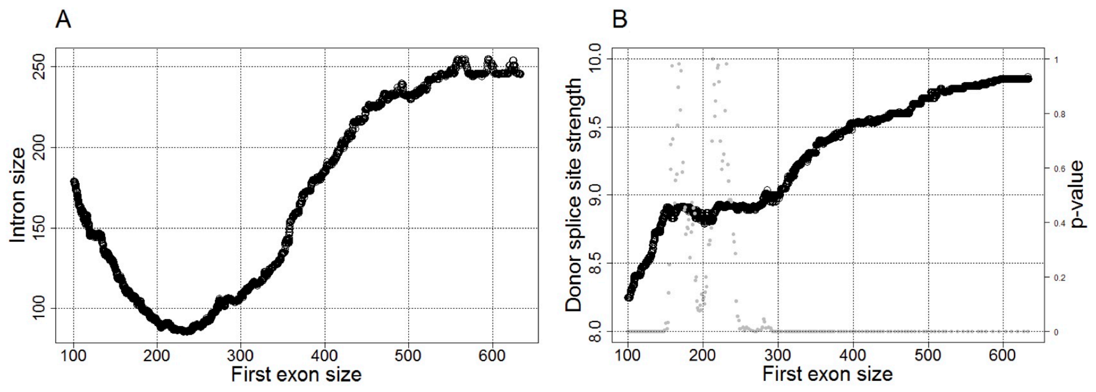

Fig. S5 [*D. yakuba*]. Relationship between first exon size (in nucleotides) and (A) first intron size or (B) strength of first-intron donor splice site. Data were ranked according to the X-axis variable and subsequent medians of 2,000 observations (step size of 1) for X- and Y-axis variables were estimated and plotted. B also shows the variations in the statistical significance of the positive association between the strength of the first donor splice site and its distance from the CBC (approximated by the first exon size). *P* values are represented by grey dots.
