# Supplementary material for: mRNA-Associated Processes and Their Influence on Exon-Intron Structure in Drosophila melanogaster
Source: G3 (Bethesda). 2016 Mar 28;6(6):1617–26. doi: 10.1534/g3.116.029231 (PMC4889658; doi:10.1534/g3.116.029231)
Supplement: Supplemental Material [file supp_g3.116.029231_FigureS6.pdf]

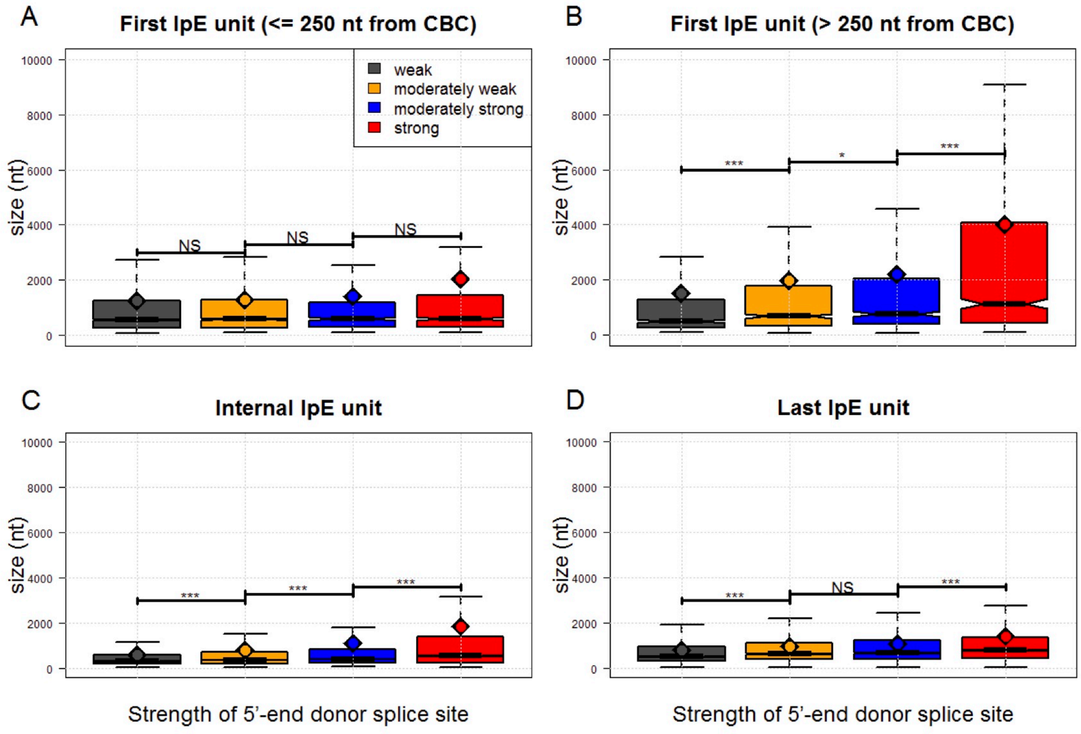

Fig. S6 [*D. yakuba*]. Relationship between the size of an Intron plus its next Exon (IpE) units in nucleotides (nt) and the strength of the associated 5'-end 5'ss, according to intragenic position. First IpE units are separated based on their distance from the CBC (*i.e.*, ≤ 250 nt and > 250 nt) and the strength of the associated 5'ss is divided into quartiles (weak ≤ 7.7 < moderately weak ≤ 9.6 < moderately strong ≤ 11.0 < strong). Median and mean strength values are illustrated with horizontal black bars and full diamonds, respectively. NS and asterisks over the bars describe the significance (or lack thereof) of statistical differences (* = p<0.05; **= p<0.01; ***= p<0.001), respectively.
