# Supplementary material for: mRNA-Associated Processes and Their Influence on Exon-Intron Structure in Drosophila melanogaster
Source: G3 (Bethesda). 2016 Mar 28;6(6):1617–26. doi: 10.1534/g3.116.029231 (PMC4889658; doi:10.1534/g3.116.029231)
Supplement: Supplemental Material [file supp_g3.116.029231_FigureS7.pdf]

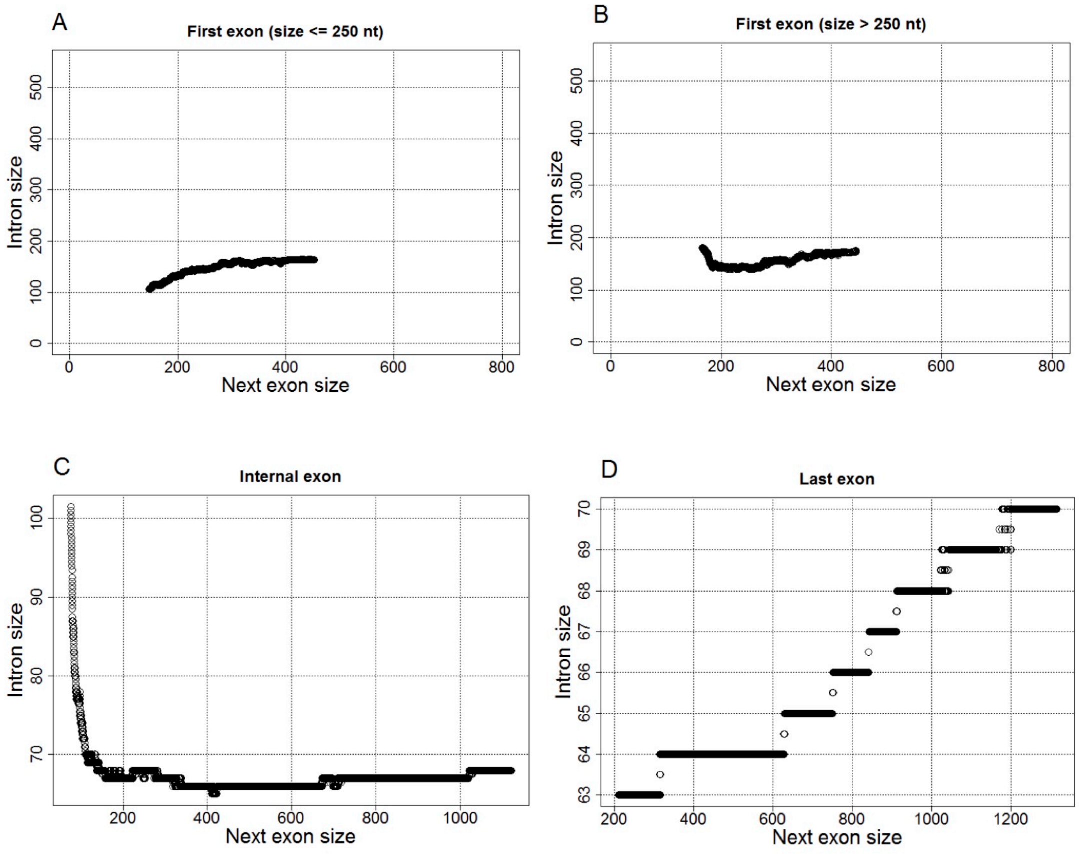

Fig. S7 [*D. yakuba*]. Relationship between the sizes of first cap-proximal and cap-distal introns (A, B), internal introns (C), and last introns (D) and their next exon. Data were ranked according to the X-axis variable and subsequent medians of 2,000 observations (step size of 1) for X- and Y-axis variables were estimated and plotted.
