# Supplementary material for: mRNA-Associated Processes and Their Influence on Exon-Intron Structure in Drosophila melanogaster
Source: G3 (Bethesda). 2016 Mar 28;6(6):1617–26. doi: 10.1534/g3.116.029231 (PMC4889658; doi:10.1534/g3.116.029231)
Supplement: Supplemental Material [file supp_g3.116.029231_FigureS8.pdf]

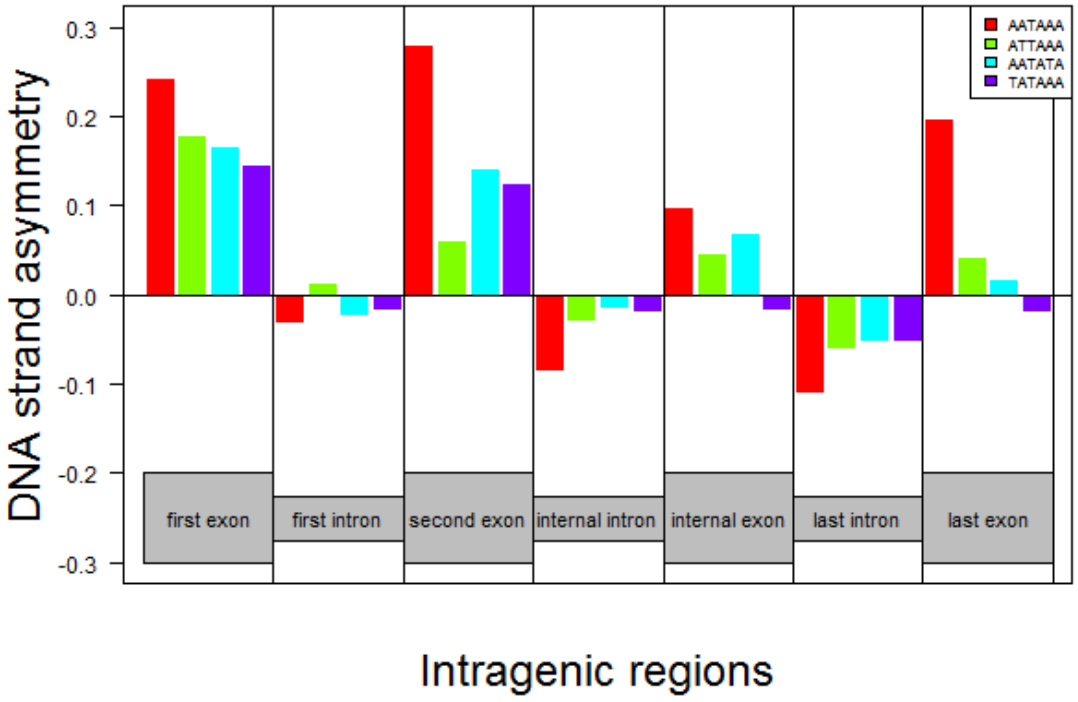

Fig. S8 [*D. yakuba*]. DNA strand asymmetry (DSA) of the canonical (strong) polyadenylation motif AATAAA and the putative polyadenylation motifs ATTAAA, AATATA, and TATAAA. DSA values are estimated separately for (first, second, internal, and last) exons and (first, internal, and last) introns.
