# Supplementary material for: mRNA-Associated Processes and Their Influence on Exon-Intron Structure in Drosophila melanogaster
Source: G3 (Bethesda). 2016 Mar 28;6(6):1617–26. doi: 10.1534/g3.116.029231 (PMC4889658; doi:10.1534/g3.116.029231)
Supplement: Supplemental Material [file supp_g3.116.029231_TableS2.pdf]

Table S2. Degree of DNA strand asymmetry (DSA) of the polyadenylation AATAAA motif and the donor splice site-like GGTAAG motif in *D. melanogaster*'s (internal and peripheral) introns with weak (≤7.7; ≤8.1) and strong (>11.0; >11.7) 5'ss and 3'ss respectively.

	**5'ss**		**3'ss**	
**Splice site strength**	**DSA**$_{AATAAA}$	**DSA**$_{GGTAAG}$	**DSA**$_{AATAAA}$	**DSA**$_{GGTAAG}$
Weak	-0.114	-0.242	-0.073	-0.315
Strong	-0.069	-0.213	-0.068	-0.196
