# Supplementary material for: mRNA-Associated Processes and Their Influence on Exon-Intron Structure in Drosophila melanogaster
Source: G3 (Bethesda). 2016 Mar 28;6(6):1617–26. doi: 10.1534/g3.116.029231 (PMC4889658; doi:10.1534/g3.116.029231)
Supplement: Supplemental Material [file supp_g3.116.029231_TableS3.pdf]

Table S3 [*D. yakuba*]. Summary statistics on the surveyed *D. yakuba* introns partitioned according to their relative intragenic position. The first and the last introns may reside in untranslated or coding regions. Sizes are expressed in nucleotides (nt). Estimates of 5'ss and 3'ss strength are calculated after excluding introns smaller than 32 nt (see Materials and Methods).

	**First**	**Internal**	**Last**
**Number of observations**	7,137	18,978	7,175
**Average (median) Intron Size**	1,504 (146)	760 (68)	343 (65)
**Average (median) Exon Size**	348 (237)	373 (211)	743 (520)
**Average (median) 5'ss strength**	8.7 (9.0)	9.3 (9.8)	9.2 (9.6)
**Average (median) 3'ss strength**	9.4 (9.7)	9.6 (9.9)	10.0 (10.2)
