# Supplementary material for: mRNA-Associated Processes and Their Influence on Exon-Intron Structure in Drosophila melanogaster
Source: G3 (Bethesda). 2016 Mar 28;6(6):1617–26. doi: 10.1534/g3.116.029231 (PMC4889658; doi:10.1534/g3.116.029231)
Supplement: Supplemental Material [file supp_g3.116.029231_TableS4.pdf]

Table S4 [*D. yakuba*]. Kendall's tau correlation coefficients that describe the strength of the association between the sizes of first, internal, or last introns and the quality of their corresponding 5'ss or 3'ss. All the corresponding *P*-values are < 0.001.

**Intron position**	**5'ss**	**3'ss**
First (5'-most)	0.076	0.178
Internal	0.170	0.034
Last (3'-most)	0.111	0.042
